# The Experimental Rate Constant of the *S*^+^(^2^*D*) + *H*_2_ Reaction

**DOI:** 10.1021/acsearthspacechem.4c00391

**Published:** 2025-02-17

**Authors:** Alexandre Zanchet, Jia Lei Chen-Qiu, Pascal Larregaray, Laurent Bonnet, Claire Romanzin, Nicolas Solem, Roland Thissen, Christian Alcaraz

**Affiliations:** † Instituto de Física Fundamental, CSIC, Serrano 123, Madrid 28006, Spain; ‡ Departamento de Química Inorgánica, Universidad Autonoma de Madrid, Madrid 28049, Spain; § Université de Bordeaux, CNRS, Institut de Sciences Moléculaire, UMR 5255, 351 cours de la libération, Talence 33405, France; ∥ CNRS, Institut de Chimie Physique, Université Paris-Saclay, UMR 8000, Orsay 91405, France; ⊥ Synchrotron SOLEIL, L’Orme des Merisiers, Saint Aubin, Gif-sur-Yvette 91192, France

**Keywords:** sulfur cation, electronic excitation, rate constant

## Abstract

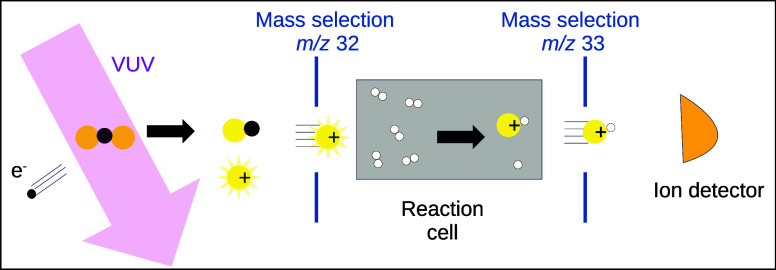

Endothermic reactions such as *S*^+^(^4^*S*) + *H*_2_ are not expected to play a significant role in the chemistry of the interstellar medium (ISM). However, in some specific environments, such as photon-dominated regions (PDR), UV radiation may catalyze the reaction by providing enough internal energy to reactants to overcome endothermicity. For instance, it was recently shown that the vibrational excitation of H_2_ greatly enhances the reactivity of C^+^ and S^+^ with H_2_, explaining the presence of their respective hydrides CH^+^ and SH^+^ in these regions. However, vibrational excitation of H_2_ is not a unique way to enhance the reactivity by UV radiation. Electronic excitation is an alternative way to effectively inject a huge amount of internal energy into the system, thus favoring reactivity. In this work, we will address how electronic excitation of the sulfur cation can strongly enhance the production of SH^+^. This is done by measuring experimentally the cross section of the title reaction for collision energies from 50 meV up to several eV and comparing the results with theoretical predictions in the 0.001–3 eV range. The reaction cross section is then used to derive the rate constant for a wide range of temperatures.

## Introduction

In the last decades, laboratory astrophysics has become essential for the interpretation of the observations made in the interstellar medium (ISM). In particular, the recent growth of the field of astrochemistry is mainly attributed to how Earth-based laboratory studies of chemistry in extreme conditions have allowed us to gather new insights into our understanding of the cosmos.^1−3^ A clear example of this interplay between laboratory astrophysics and astronomy can be found at Leiden Observatory, and in particular through the work of Harold Linnartz, to whom this work is dedicated. During his career, he focused on understanding the chemical complexity under the extreme conditions of space, and he could demonstrate the relevance of fundamental physical chemistry processes in the understanding of chemistry in the ISM. In particular, to be mentioned is the application of molecular spectroscopy to determine the carriers of diffuse molecular bands in the ISM^4^ as well as his pioneering research on chemistry and photochemistry in ices, showing these processes were a key ingredient in the complexification of molecules in space.^5−7^

Another important aspect of laboratory astrophysics is the study of chemical reactions under extreme conditions in the gas phase. Although part of the chemistry in the ISM is thought to occur on ices and grains, gas-phase chemical reactions may also be very efficient for the production of some species. There are several examples of reactions in the gas phase involving ions and/or radicals, which can occur without an activation barrier and become faster at low temperatures.^8^ And even in the case of endothermic reactions, in regions under high UV radiation such as photon dissociation Regions (PDRs), UV photons or electrons may provide enough internal energy to the system to considerably accelerate chemical reactions. For instance, *H*_2_ has recently been observed with a significant amount of vibrational energy in the Orion Bar PDR due to UV pumping.^9^ Considering that each vibrational quantum of *H*_2_ brings a considerable amount of energy to the system, vibrational excitation of *H*_2_ greatly enhances its reactivity and may promote endothermic reactions, as has been shown for *C*^+^ + *H*_2_,^10^*S*^+^ + *H*_2_,^11−13^ or *O* + *H*_2_,^14,15^ among others.

However, vibrational excitation is not the only way to transfer energy from a photon to a molecular system. During an electronic transition, the photon energy is directly transferred to the electrons of an atom or a molecule, typically providing more internal energy than vibrational excitation, for which energy is located in nuclear motion. While the difference in energy between *H*_2_(v = 0) and *H*_2_(v = 1) is ≈0.5 eV, the difference in energy between the ground and first excited electronic state of sulfur cation *S*^+^(^4^*S*) and *S*^+^(^2^*D*) is ≈1.83 eV, which corresponds to an internal energy larger than three vibrational quanta of *H*_2_. It is therefore expected that *S*^+^(^2^*D*) will have considerably enhanced reactivity compared to *S*^+^(^4^*S*). Actually, the high reactivity of *S*^+^(^2^*D*) with *H*_2_ has already been observed and reported in the literature by Tichý et al.^16^ and was confirmed later on by Stowe et al.^17^ who studied the kinetic energy dependence of this reaction with a mixture of ground and excited sulfur cation, showing that excited S^+^ reacts with no barrier with H_2_. There is additionally several evidence of the presence of *S*^+^(^2^*D*) in the Orion Bar,^18−20^ in planetary nebulae^21,22^ or in planetary and moon plasma torus,^23,24^ and considering the relatively large lifetime of this excited ion (≈1500 *s* and 5000 *s* for ^2^*D*_3/2_ and ^2^*D*_5/2_, respectively),^25^ its reactivity with *H*_2_ may be relevant for sulfur chemistry in the ISM.

In this work, we present an experimental and theoretical study of the *S*^+^(^2^*D*) + *H*_2_ reaction with the aim of providing the reaction rate constant of this reaction as a function of temperature. The work is structured as follows: in the Methods section, we will describe the experiment and the theoretical methodology employed, then the results will be presented and discussed before the conclusion.

## Methods

### Experiment

The ion–molecule reaction *S*^+^(^2^*D*)+*H*_2_ was studied with the Guided Ion Beam setup CERISES^26,27^ attached to the DESIRS beamline^28^ at the French synchrotron SOLEIL. The setup is based on a sequence of 4 radio frequency (RF) devices: quadrupole-octopole-octopole-quadrupole (QOOQ). This allows first the mass selection (Quad 1) of parent cations produced in the source, then to control the collision energy (Oct 1) between the ion colliding with the target gas, and finally, to select in mass (Quad 2) both the unreacted parent ions (*S*^+^) and product ions (*SH*^+^) before their detection. Absolute reaction cross sections can then be extracted from the measured ion yields and the absolute pressure of the target gas, which are set to ensure the single-collision regime. The effective cell length is obtained using the calibration reaction Ar^+^ + D_2_ → ArD^+^ + D.^29^ Additional technical details on the experiment can be found in our previous work on the reactivity of sulfur cation in the ground state (^4^*S*) with H_2_.^30^

In order to generate the sulfur cation in its excited state (^2^*D*), VUV synchrotron radiation of 17.7 eV has been employed to produce the dissociative photoionization of the precursor *CS*_2_. In the experiment, the time-of-flight between the production of the parent ion in the source and their reaction in the cell is of the order of 100 μs, which is several orders of magnitude lower than the lifetime of *S*^+^(^2^*D*) of thousands of seconds,^25^ so the amount of excited ion will remain constant during the whole experiment. In addition to *S*^+^(^2^*D*), dissociative photoionization of *CS*_2_ at 17.7 eV also produces *S*^+^(^4^*S*), which cannot be discriminated in the experiment. As a consequence, the absolute cross section measured in the experiment is associated with a mixture of *S*^+^(^4^*S*) and *S*^+^(^2^*D*), whose proportions are not exactly known. Nevertheless, since we know the absolute cross section of *S*^+^(^4^*S*) + *H*_2_ from our previous work,^30^ and a relatively good estimate of the reaction cross section *S*^+^(^2^*D*) + *H*_2_ can be obtained from theory, it is possible to trace back the respective quantum yield of *S*^+^(^4^*S*) and *S*^+^(^2^*D*) arising from *CS*_2_ irradiated at 17.7 eV.

### Theory

In the experiment, *S*^+^(^4^*S*) and *S*^+^(^2^*D*) are products of the dissociative photoionization of *CS*_2_ at 17.7 eV but cannot be discriminated. As a consequence, the measured cross section corresponds to the ponderated average of *S*^+^(^4^*S*) + *H*_2_ and *S*^+^(^2^*D*) + *H*_2_ cross sections:
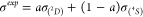
1with *a* being the fraction of *S*^+^(^2^*D*).

Since  is already known from our previous work,^30^ it is possible to derive a good estimation of *a* if we can provide a good theoretical estimation of . Once *a* is determined, the absolute experimental cross section  can then be recovered.

**Figure 1:**
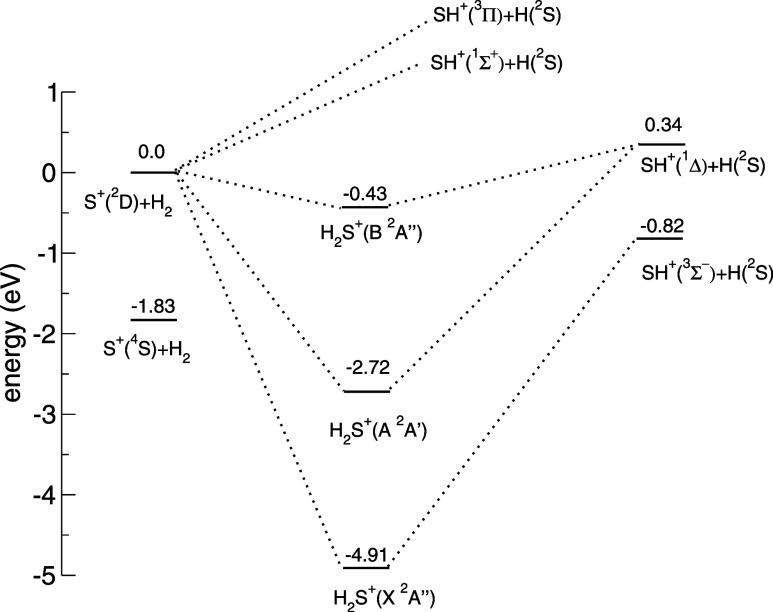
Adiabatic correlation diagram of the doublet states of *H*_2_*S*^+^ according to the PES employed in this work. The relative energy of the ground state of the *S*^+^ + *H*_2_ channel is also shown. Vibrational zero point energies (ZPE) are not considered in this diagram.

To provide an estimation of , in this work, we will study the reaction dynamics of *S*^+^(^2^*D*) + *H*_2_ using Quasi-Classical Trajectories (QCT). In the experiment, the *H*_2_ diluted gas is around 300 K, so it cannot be vibrationally excited and only a few rotational levels are populated. Since a small rotational excitation is not expected to play a significant role in the reaction, all QCT calculations were performed considering *H*_2_ in its rovibrational ground state. In this work, the origin of energies has been set to the *S*^+^(^2^*D*) asymptotic channel so that the total kinetic energy of the system corresponds to the total energy available. This way, it is easy to understand that all the negative regions of the potential can be accessed even at extremely low collision energies. As illustrated in the correlation diagram of the *H*_2_*S*^+^ triatomic system depicted in Figure 1, ignoring spin–orbit couplings, *S*^+^(^2^*D*) consists of five degenerated electronic states, two of symmetry ^2^*A*′ and 3 of symmetry ^2^*A*″ in the *C*_*s*_ representation. For each of them, the interaction with molecular hydrogen will be different, so during the collisional process, when *S*^+^ gets close to *H*_2_, the 5-state degeneracy of the atom is lifted. Since the collision can occur on any of the five potential energy surfaces (PES) with the same probability, the partition function is 1/5 for all the states. The total reaction cross section of *S*^+^(^2^*D*) + *H*_2_ is thus obtained as the average of the individual cross sections of the respective electronic states:
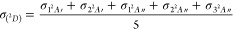
2

Since the electronic states 2^2^*A*′ and 3^2^*A*″ exhibit repulsive interaction in the reactant channel *S*_+_ + *H*_2_ and both correlate adiabatically to high-lying electronic states of the product channel *SH*^+^ + *H*, they are not expected to produce a reaction, and their contribution to the reactive cross section will be zero. Eq 2 thus becomes
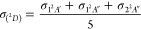
3

As a first approximation, in this work, the couplings between the three considered electronic states will not be considered, and the three state-specific cross sections , , and  will be obtained by studying the reaction dynamics on their respective PESs. The PESs considered in this work are the same as in ref (30) for the 1^2^*A*’ and 1^2^*A*” states. The PES of 2^2^*A*″ was obtained by fitting the corresponding MRCI points, which were calculated together with the MRCI points of the other two states. A more detailed description of the topography of this new PES will be described in a future work as the characteristics of the electronic structure of *H*_2_*S*^+^ are out of the scope of this work.

The QCT methodology was applied as proposed by Karplus et al.^31^ Briefly, it consists of describing the nuclear motion trajectories for a great ensemble of well-sampled initial conditions (positions and momenta) by integrating the classical Hamilton’s equation of motion. For the sampling of initial conditions, we followed the same strategy as for the neutral equivalent reaction *S*(^1^*D*) + *H*_2_,^32^ with the only difference being that the initial distance was increased to 20 Å in the case of the cation, while 15 Å was considered for the neutral.

One of the drawbacks of the QCT method is that classically, energy is not quantized, and there are no restrictions to prevent vibrational energy leaking. This may be problematic when collisions occur in the quantum regime, i.e., when a few states are available to reactants or products. The Gaussian binning, which is widely discussed in the literature,^33−42^ has been proposed to overcome this approximation. It consists of assigning Gaussian statistical weights to trajectories in such a way that the closer the final vibrational action *v* is to integer values, the larger the weight. However, Gaussian weights should not be assigned to nonreactive paths along which the internal motion of the reagents evolves adiabatically throughout the collision. In such a case, an adiabaticity correction must be introduced in the treatment. Details are given in refs. (32,36) and (40).

In this work, we apply the Normalized Gaussian Binning with Adiabatic Correction (NGBAC) following the methodology of ref. (32), which was found to lead to cross sections in very good agreement with quantum scattering calculations for the *S* + *H*_2_ collision. Within the NGBAC, the *J*-resolved reaction probability reads as follows:
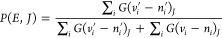
4where the Gaussian weights are computed on the reactive and nonadiabatic nonreactive trajectories with for a given trajectory *i*, *v/v’* refers to the final vibrational action of reactants/products, *n/n’* is the closest integer value to *v/v’*, and . The semiclassical theory of molecular collisions tells us that in the classical limit, i.e., when *ℏ* is made to tend to 0 in the quantum mechanically exact expression of state-to-state reaction probabilities, trajectories must be assigned delta functions δ*(v – n)* (δ*(v’ – n’)*).^40^ Numerically, however, the probability that a given trajectory ends with *v* (*v’*) exactly equal to *n* (n') is nearly 0. Consequently, the δ functions were replaced by Gaussian weights normalized to unity with a nonzero width well below the unit spacing between two consecutive quantum states. The value ϵ = 0.06 leads to a width of one-tenth, which requires running ten times more trajectories for the same statistical bias as the standard QCT method. The quantization of the vibrational motion is then satisfactory, while the increase in computing time remains reasonable. In the calculations, the maximum values of *J*, the total angular momentum, vary from 12 to 128, depending on the electronic state and collision energy. As we fix the number of trajectories at each *J* (between approximately 6000 and 40000), the total number of trajectories differs for each calculation, being always greater than 450000. For the 1^2^*A″* electronic state, the maximum values of the computed classical vibrational and rotational actions for the products are *v*′ = 3.2 and *j′* = 31.0 at 0.005 eV collision energy (*v′* = 12.7 and *j′* = 66.3 at 3 eV). For the 1^2^*A′* and 2^2^*A*″ states, they vary from *v′* = 0.13 and *j′* = 12.8 at 0.3 eV to *v′* = 10.0 and *j*′ = 54.0 at 3 eV. To assess the convergence of the computed cross sections, 4 independent calculations have been carried out at 0.005, 1.6, and 3 eV collision energies for the 1^2^*A*″ state and 0.3, 1.6, and 3 eV for the 1^2^*A′* and 2^2^*A*″. The dispersions of the predicted cross sections were less than 1% in each case.

The QCT cross section is recovered from *P(E,J)* using
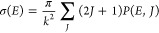
5with k as the wave vector. Finally, the rate constant can be obtained directly from the cross section as^31^
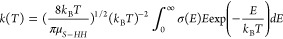
6with μ_*S–HH*_ as the reduced mass of *S*^+^ + *H*_2_ and *k*_*B*_ as the Boltzmann constant. This latter equation can be applied independently to any cross section, whether measured experimentally or calculated theoretically.

## Results and Discussion

**Figure 2:**
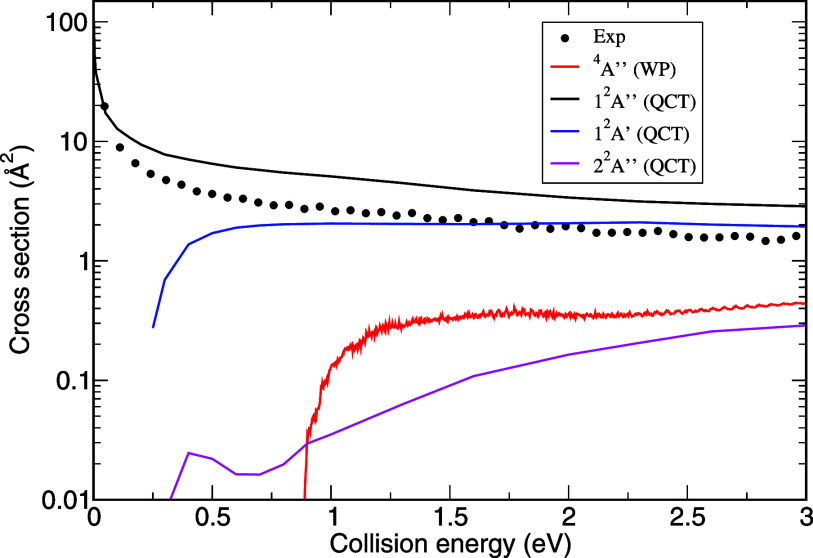
*S*^+^ + *H*_2_ → *SH*^+^ + *H* cross section. Black dots are the measured cross sections associated with the *S*^+^(^4^*S*, ^2^*D*) mixture arising from *CS*_2_ irradiated at 17.7 eV. The red curve represents the theoretical cross section associated with *S*^+^(^4^*S*) from ref. (30). The black curve represents the cross section of the 1^2^*A*″ state. The blue curve represents the cross section of the 1^2^*A′* state. The magenta curve represents the cross section of the 2^2^*A*″ state. The partition function factor  is already considered for the cross sections of the doublet states.

Figure 2 shows the theoretical state-specific cross sections calculated up to 3 eV, together with the absolute cross section of the mixture *S*^+^(^4^*S*, ^2^*D*) colliding with *H*_2_ that has been measured experimentally. At low collision energies, when couplings are neglected, only the 1^2^*A*″ state contributes to the cross section. This is in accordance with the correlation diagram shown in Figure 1 where it appears that it is the only state for which the reaction is exothermic. This feature, as well as the fact that this state does not present any barrier to reaction, is consistent with previous electronic structure calculations^17,43^ and explains why the reaction cross section decreases strongly with collision energy, as previously observed.^17^ This state clearly appears as the most reactive and provides the major contribution to the total cross section. The threshold of reaction appears at higher collision energies for the 1^2^*A*′ and 2^2^*A*″. These two states correlate to *SH*^+^(^1^Δ) and share the same threshold of 0.25 eV when accounting for the ZPE of reactants (0.27 eV) and products (0.16 eV) in addition to the 0.34 eV endothermicity given by the electronic potential, which corresponds to the difference of energy between *S*^+^(^2^*D*) + *H*_2_*(v* = *0, j* = *0)* and *H + SH*^+^(^1^Δ)*(v = 0, j = 0)*, meaning that none of these states present a reaction barrier larger than the endothermicity. Nevertheless, the 2^2^*A*″ state is considerably less reactive than the 1^2^*A*′, which is attributed to the fact that in its *B* state, the *H*_2_*S*^+^ complex exhibits considerably lower stability (−0.43 eV) than the ground state (−4.91 eV) and the A state (−2.72 eV). Finally, the threshold of reaction is even higher for *S*^+^(^4^*S*) as the reaction only opens at 0.89 eV.^30^ However, above the threshold, for an equivalent proportion of *S*^+^(^4^*S*, ^2^*D*), the contribution of the ^4^*A″* state to the cross section would be larger than the 2^2^*A*″ contribution.

**Figure 3:**
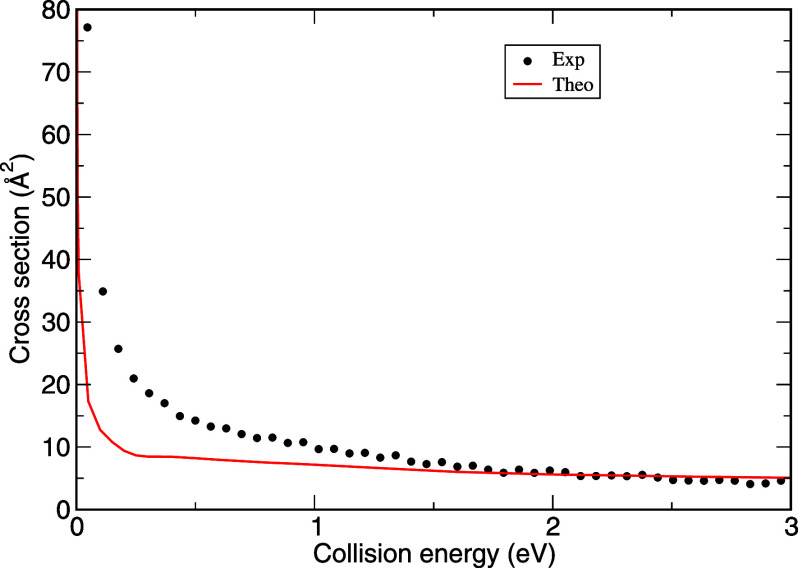
*S*^+^(^2^*D*) + *H*_2_ → *SH*^+^ + *H* cross section. The black dots represent the experimental cross section derived as , considering *a* = 0.26. The red curve corresponds to the QCT cross section, summing all state-specific cross sections with the appropriate ponderation.

The fact that the experimental cross section is lower than the 1^2^*A*″ cross section reflects that a significant proportion of *S*^+^ ions are in their electronic ground state (^4^*S*). Considering the theoretical cross sections of the four states depicted in Figure 2 and eq 3, *a* can be directly determined from eq 1 as . To reduce the impact of noise on the estimation of *a*, it is convenient to consider the average value obtained over a wide range of collision energies. In principle, the average could be performed over the whole range of energy, but there are physical considerations, suggesting that this is not the best choice. The total fragmentation channel *S*^+^(^4^*S*) + *H*(^2^*s*) + *H*(^2^*S*) opens around 2.65 eV on the 1^2^*A*″ state, with low efficiency around the threshold, reaching a probability of 3% for collision energies of 3 eV. Above 3 eV, this competing mechanism becomes even more significant and may perturb the dynamics in such a way that cannot be properly captured by the theoretical description since the couplings in the total fragmentation asymptotic region have never been explored for this system. Therefore, another competing mechanism may perturb the theoretical description of the reaction dynamics. And at low collision energies, couplings between electronic states (neglected in this work) are expected to play a more important role than at higher collision energy. This will be discussed in more detail later. We thus decided to restrict the average value of *a* in the [2–3] eV energy range, which is found to be *a* = 0.26, indicating a fraction of 26% of *S*^+^(^2^*D*) and 74% *S*^+^(^4^*S*). The resulting experimental  and its theoretical counterpart are shown in Figure 3.

**Figure 4:**
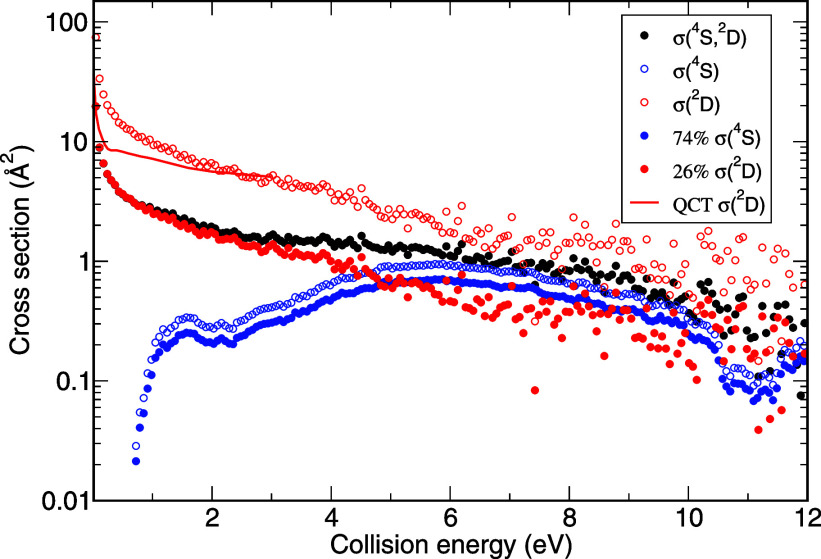
Measured *S*^+^(^4^*S*, ^2^*D*) + *H*_2_ → *SH*^+^ + *H* experimental cross section at 17.7 eV (black circle) together with the pure *S*^+^(^4^*S*) + *H*_2_ → *SH*^+^ + *H* experimental cross section^30^ (blue open circle) and the *S*^+^(^2^*D*) + *H*_2_ → *SH*^+^ + *H* estimated in this work (red open circle). The respective contributions, considering that 74% of cations are in state (^4^*S*) (red circle) and 26% of cations are in state (^2^*D*) (blue circle), are also shown.

We should point out that  was found to be in very good agreement with the experimental cross section,^30^ so the experimental cross section of *S*^+^(^4^*S*) can also be used instead of the theoretical one. Since the proportion of *S*^+^(^4^*S*, ^2^*D*) does not depend on the collision energy and the experimental  and  were measured in a wider range of collision energy, we can thus extract the experimental  for the whole collision energy range as
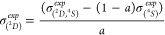
7with *a* = 0.26. The results are depicted in Figure 4. It is interesting to observe that above 6 eV, the ponderated contributions  and  are nearly equivalent. This is not the case in the energy range of 3–6 eV where both  and  vary significantly. We can thus appreciate how the estimated fraction of *S*^+^(^4^*S*, ^2^*D*) derived in the 2–3 eV range satisfactorily reproduces the measured experimental signals in the whole energy range, thus validating the approach of considering the 2–3 eV interval and the assertion of *a*.

This is not exactly the case if the theoretical  is considered instead of the experimentally derived one. While the agreement between theory and experiment is good for larger collision energies (up to 3 eV), increasing discrepancies are observed when collision energies get smaller, as it appears clearly in Figure 4. This suggests that, as for *S*^+^(^4^*S*), spin–orbit couplings between the different electronic states may play an important role in the case of *S*^+^(^2^*D*).^30^ This can be rationalized by the fact that the lifetime of the stable intermediate complex in the 1^2^*A*′ PES increases at low collision energy. While at 3 eV, the mean time of the trajectories is of the order of hundreds *fs*, at 0.3 eV, it increases to the *ps* time scale and reaches tens of *ps* at 0.001 eV collision energy. These huge lifetimes at low collision energies thus favor population transfer toward the 1^2^*A*″ state, on which products can be reached even at low energy.

In addition to spin–orbit couplings, the possibility that the nonadiabatic couplings arising from the conical intersection between the 1^2^*A*′ and 2^2^*A*′ states^43^ and Renner–Teller couplings between 1^2^*A*′ and 1^2^*A*″^44^ cannot be discarded either, increasing even more the possibilities of a population transfer. In the case of *S*^+^(^4^*S*), the nonadiabatic couplings were not found relevant for the reaction, probably due to the fact that both conical intersection and linear configurations of *H*_2_*S*^+^ lie at relatively high energies above the reactant channel *S*^+^(^4^*S*) + *H*_2_. This is not the case for *S*^+^(^2^*D*) as both the conical intersection and Renner–Teller^44^ region are located below the *S*^+^(^2^*D*) + *H*_2_ reactant channel and can thus be accessed, even at very low collision energies. A study including these couplings will thus be necessary to improve the theoretical description of this reaction and refine the estimation of the *S*^+^(^4^*S*, ^2^*D*) fraction. Nevertheless, even if the estimation of *a* can probably be further improved, the consistency observed in Figure 4 when comparing the experimental cross sections suggests that the estimation of 26% of *S*^+^(^2^*D*) in the source should be very close to the true fraction, and thus,  experimentally derived in this work is expected to be quite accurate.

This is also supported by the good comparison of the present results with those reported by Stowe et al.^17^ obtained with a similar experimental setup and for which the same precursor (*CS*_2_) was employed. There are, of course, some differences between the experiments, as in their work, sulfur cations were generated by electron impact ionization instead of photoionization in our setup. In their work, several electron energies were employed, including larger energies leading to the production of S^+^(^2^*P*) in some of the mixtures. Combining the measurements made with different proportions of ground and excited cations, they were able to deduce the cross sections associated with a mixture of excited *S*^+^(^2^*D*) and *S*^+^(^2^*P*), but not shown in the case of *H*_2_. From the different cross sections, they could then derive with great accuracy the cross section associated with *S*^+^(^4^*S*), for which the comparison with theory was very good.^30^ Although a direct quantitative comparison cannot be made between the two experiments because the proportions of *S*^+^(^2^*P*) are not known in their experiment, a quick qualitative comparison still shows that the present *S*^+^(^4^*S*,^2^*D*) cross section for the *H*_2_ target exhibits similar energy dependence to the one associated with *S*^+^(^4^*S*, ^2^*D*, ^2^*P*) for the *D*_2_ target presented in Figure 1 of Stowe et al.^17^

In order to provide the rate constant of the *S*^+^(^2^*D*) + *H*_2_ reaction, we considered the experimental cross section. To solve the integral in eq 6, we fitted the experimental  to a modified Langevin-like functional form σ = *A.E*^–1*/n*^ in the 0–3 eV interval. This expression, which lacks physical meaning, was used for two purposes: to better fit the data by gaining flexibility in letting the parameter *n* vary and to give an estimation on how much the cross section deviates from the Langevin behavior, given by integer values of *n*. The best fit was obtained with A = 9.4525 and *n* = 1.7365, showing deviation from the Langevin behavior (which would correspond to *n* = 2). The resulting  values are tabulated in Table 1 for temperatures in the 1–2500 K interval.

**Table 1:** Rate Constant for the *S*^+^(^2^*D*) + *H*_2_ Reaction as a Function of the Temperature

T (*K*)	(cm^3^.·s^–1^)
1	1.940 × 10^–9^
10	1.629 × 10^–9^
20	1.545 × 10^–9^
30	1.499 × 10^–9^
40	1.459 × 10^–9^
50	1.442 × 10^–9^
60	1.422 × 10^–9^
70	1.405 × 10^–9^
80	1.391 × 10^–9^
90	1.379 × 10^–9^
100	1.368 × 10^–9^
200	1.298 × 10^–9^
300	1.258 × 10^–9^
400	1.231 × 10^–9^
500	1.211 × 10^–9^
600	1.194 × 10^–9^
700	1.180 × 10^–9^
800	1.168 × 10^–9^
900	1.158 × 10^–9^
1000	1.149 × 10^–9^
1500	1.114 × 10^–9^
2000	1.090 × 10^–9^
2500	1.071 × 10^–9^

As we saw previously, and as expected for a barrierless ion/neutral exothermic reaction, the cross section increases at low temperature, and similarly, the rate constant also increases for decreasing temperature. Interestingly, it appears that the experimental cross section  do does follow closely a typical Langevin behavior expected for this kind of reaction. But when the  contribution is subtracted, the Langevin behavior is lost, and the resulting  cross section decreases faster than Langevin, explaining why the rate constant increases at low energy. It is instructive to compare the obtained rate constants with the Langevin constant *k*_*L*_. Considering the polarizability of molecular hydrogen  = 0.805 Å^3^,^45,46^ we find *k*_L_ = 1.526 × 10^–9^ cm^3^/s, quite close to the rate constant at 10–30 K. Of course, the precision of the rate coefficients derived here depends mainly on the determination of the parameter *a* and the error of the fit of the cross section to the analytical form employed. There are several experimental sources of error that may affect the determination of *a*, like the statistical noise in the measurements, the thermal broadening of hydrogen gas, and the energy dispersion of the ion beam. There are also several theoretical sources of error, such as the accuracy of the PESs employed, the intrinsic limitation of the QCT methods, the neglect of couplings, and the choice of the energy range considered for the estimation of *a*. Considering all these factors, it is hard to provide an accurate estimation of the uncertainties, but we believe that they should not exceed 50% of error for the fitted  employed in eq 6, and consequently, we estimate less than 50% error on the rate coefficients provided in this work.

It is interesting to compare in which form the additional internal energy is more efficient in promoting the reaction. By comparing the rate constant of *S*^+^(^2^*D*) + *H*_2_ to those of *S*^+^(^4^*S*) + *H*_2_(*v*) of ref. (13), it appears that the rate constants for the sulfur cation in the excited state with hydrogen are larger than those of the ground-state cation with vibrationally excited H_2_(*v* = 5), particularly at low temperatures. If we consider that the electronic excitation of *S*^+^ provides an additional 1.8 eV to the internal energy of the system, while 5 quanta of vibration of *H*_2_ provide ≈2.3 eV to the system, it appears clearly that in the case of the title reaction, the electronic excitation of the cation is more effective than the vibrational excitation of *H*_2_.

## Conclusion

In this work, we present the experimental rate constant of the reaction *S*^+^ + *H*_2_ → *SH*^+^ + *H* over a wide range of temperatures. This rate constant is obtained by combining, in an ingenious way, both experimental and theoretical data as follows: the reactants are *H*_2_ and a mixture of sulfur cations in two different electronic states, (^4^*S*) and (^2^*D*), whose proportions are given by the photodissociative ionization of *CS*_2_ at 17.7 eV. By combining the cross section measured from this mixture with *H*_2_, the known cross section of *S*^+^(^4^*S*) + *H*_2_ → *SH*^+^ + *H* and a theoretical estimation of the cross section associated with *S*^+^(^2^*D*) over a carefully chosen collision energy range, it is possible to infer the respective proportions of *S*^+^(^4^*S*)/*S*^+^(^2^*D*). Knowing the proportions and the cross section of *S*^+^(^4^*S*) + *H*_2_, the experimental cross section of *S*^+^(^2^*D*) could be recovered over the whole range of energies, allowing the determination of the state-specific rate constant of the sulfur cation in its excited state.

It appears that the reactivity of the cation is significantly enhanced when it is electronically excited, with an increase in reactivity larger than the one obtained from the vibrational pumping of *H*_2_ for an equivalent amount of internal energy injected into the system. Moreover, it is found that the reaction becomes faster at low temperatures, which makes it potentially relevant for sulfur chemistry in the ISM. The rate constants provided in this work are expected to be useful to astrochemistry modelers in order to improve the current knowledge of the chemical network of sulfur in the ISM as this reaction may be relevant in UV-irradiated objects such as PDRs, protoplanetary disks, higher layers of planetary atmospheres, or comets’ comas .
